# Heterogeneous impacts of work–family conflict on workforce mental health: evidence from the differential effects of work-to-family and family-to-work conflict

**DOI:** 10.3389/fpubh.2026.1832589

**Published:** 2026-05-28

**Authors:** Yuqian Wang, Jianbo Zhang, Chao Li

**Affiliations:** 1 Business School, Shandong University, Weihai, China; 2 Centre for Quality of Life and Public Policy Research, Shandong University, Qingdao, China

**Keywords:** family-to-work conflict, perceived depression, work-family balance, workforce mental health, work-to-family conflict

## Abstract

**Purpose:**

Despite extensive discussions on the negative consequences of work-family conflict, the heterogeneous impacts of WFC and FWC on perceived depression have not been systematically examined. This study aims to systematically examine the relationship between work-family conflict and perceived depression from the novel heterogeneous perspectives of work-to-family conflict (WFC) and family-to-work conflict (FWC).

**Materials and methods:**

Empirical analysis is conducted based on data from the Chinese General Social Survey.

**Results:**

WFC and FWC significantly increase depression levels, with FWC exerting a more pronounced impact. These conflicts reduce life happiness and work satisfaction, thereby increasing depression. The effect of WFC is more significant among men, individuals under 40, those with lower education level, married ones, those having children, and people with poorer economic status. Moreover, its effects are also more pronounced for those in managerial positions, with low job autonomy, performing non-cognitive work, working outside the primary labor market, without social security, and in areas with a higher competitive labor market. In contrast, FWC affects higher educated and unmarried individuals more, showing no notable differences in terms of gender, age, or managerial roles. In other aspects, the heterogeneity of FWC's impact generally aligns with that of WFC.

**Conclusion:**

These findings highlight the importance of reducing work-family conflicts to address depressive emotions and the necessity of implementing targeted measures to mitigate WFC and FWC across different populations.

## 1 Introduction

Depression, as one of the most prevalent mental health issues, has become a significant factor affecting people's overall quality of life. According to the World Health Organization (1), over 280 million people globally are affected by depression. It not only impairs cognitive function and behavioral performance but also serves as an important risk factor

for numerous serious physical illnesses (2–4), including kidney disease (5), myocardial infarction (6), diabetes (7), stroke (8), and several types of cancer (9). In severe cases, depression can further lead to suicidal tendencies (10, 11). Therefore, examining the predisposing factors of depression holds significant public health importance (1). The etiology of depression is complex and multifaceted, spanning biological, psychological, and social and environmental aspects (12, 13). Studies on the social and environmental factors primarily focus on demographic characteristics, human capital attributes, social contexts, and family traits (14–16). As research on the influencing factors of depression deepens, work-family conflict has gradually become a focal point. Work-family conflict is commonly defined as a type of inter-role conflict where participation in the work (family) role is made more challenging due to participation in the family (work) role (17).

Resource Conservation Theory's Scarcity Hypothesis holds that individuals possess finite resources that must be allocated between work and home. When individuals attempt to manage both roles simultaneously, these resource constraints intensify role conflict and amplify tensions between work and family (18, 19). Empirical evidence from diverse cultural settings, such as the Kingdom of Saudi Arabia, Italy, and Canada, has shown that work-family conflict significantly and negatively impacts various domains of individuals' lives. First, it may reduce the time spent with family members, thereby diminishing life satisfaction and happiness (20–22). Long-term work-family imbalance is closely linked to decreased marital satisfaction and increased family dissolution (23). Second, work-family conflict may lead to reduced work hours and distraction, lowering work performance and work satisfaction, as well as contributing to burnout, decreased work engagement, and limited career development (24–27). Third, it may lead to higher long-term sickness absence and increase turnover intentions, as individuals might leave their jobs to alleviate stress when struggling to balance work and family roles (28, 29).

Existing studies have extensively documented the adverse impacts of work-family conflict (30–32) and more recent research has begun to emphasize the importance of distinguishing between work-to-family conflict (WFC) and family-to-work conflict (FWC). Despite this promising progress, the literature still lacks a systematic assessment of how WFC and FWC generate heterogeneous effects on perceived depression. In addition, while work-family conflict has been shown to be positively associated with depression (30, 33–35), the underlying mechanisms are still not well established. Prior studies suggest that work-family conflict is negatively associated with life happiness and work satisfaction (21, 36, 37), and that happiness and work satisfaction are themselves negatively related to perceived depression (38). However, whether work-family conflict affects perceived depression through these two factors remains to be tested.

Against the background of the aforementioned research gaps, this study makes contributions primarily in the following aspects. First, it distinguishes between WFC and FWC, and from this new perspective, deepens our understanding of their heterogeneous impacts on perceived depression. WFC refers to the interference of work-related stressors or demands with an individual's ability to fulfill family roles. Conversely, FWC manifests as family responsibilities hindering the effective execution of work duties (39, 40). Specifically, WFC and FWC represent two forms of inter-role conflict, each rooted in a different source of role interference. WFC represents a type of conflict that stems from work-related conduct, time allocated to work, and occupational strain, all of which hinder individuals from discharging their family-related duties. In contrast, FWC constitutes another form of conflict arising from family-related conduct, time devoted to family, and familial strain, which interfere with the completion of individuals' work-related obligations. Using Chinese data, the study estimates these divergent impacts and thereby deepens our understanding of how distinct forms of conflict relate to mental health.

Second, the study clarifies the pathways through which work-family conflict affects perceived depression and how these pathways differ across demographic groups. Specifically, it treats life happiness and work satisfaction as mediating variables to test whether work-family conflict increases perceived depression by reducing these two factors. By doing so, the study not only extends existing research on the mechanisms linking work-family conflict and mental health, but also offers a more nuanced and detailed explanation of this complex relationship. In addition, by examining diverse demographic, family, work, and labor protection characteristics, this study investigates the differential impacts of WFC and FWC on depression. Thus, it further enriches our knowledge of how work-family conflict relates to perceived depression and its heterogeneity, offering valuable insights for assessing the risk of depression due to work-family conflict in different subpopulations.

The remainder of this paper is organized as follows. Section 2 introduces the data sources and variables. Section 3 presents the benchmark analysis, mechanism tests, and heterogeneity analysis. Section 4 discusses the findings of this research. Section 5 concludes the paper.

## 2 Materials and methods

### 2.1 Data resources

The data used in this study come from the Chinese General Social Survey (CGSS), which is China's earliest national, comprehensive and continuous academic survey project. Its sample covers 28 provinces/municipalities/autonomous regions in China and uses the multi-stage stratified Probability Proportionate to Size (PPS) sampling method, making it highly representative. Beyond national representativeness, CGSS offers several advantages for this study. It investigates individuals' perceived depression and information about work-family conflict, along with comprehensive factors influencing depression, facilitating the construction of the dependent variable, explanatory variable and control variables for this research. This study utilizes data from the extended module in the 2021 wave of CGSS, which includes information on work-family conflict.

### 2.2 Variables

#### 2.2.1 Dependent variables

The main dependent variable in this study is respondents' perceived depression, denoted as Depression. This variable is derived from a question in the core module of the CGSS: “To what extent do you feel depressed?” The options for this question are based on a Likert scale ranging from 1 to 5, including “1-not depressed”, “2-mildly depressed”, “3-moderately depressed”, “4-very depressed” and “5-severely depressed.” This indicator is widely used to measure the degree of self-rated depression (41). The larger the value of this variable, the higher the level of depression.

#### 2.2.2 Explanatory variables

This study measures the explanatory variable, work-family conflict, from two dimensions: work-to-family conflict (WFC) and family-to-work conflict (FWC). The indicators are derived from the following questions: “To what extent does your work interfere with your family life?” and “To what extent does your family life interfere with your work?” Respondents answer these questions on a scale of 1 to 5, representing “never interfered”, “rarely interfered”, “sometimes interfered”, “frequently interfered”, and “always interfered”. A higher score indicates a greater degree of interference from work to family or family to work, signifying a more intense work-family conflict.

#### 2.2.3 Control variables

Based on the literature regarding factors influencing perceived depression (42–44), this study includes controls comprehensively for a variety of factors affecting individuals' self-rated depression, including demographic and human capital characteristics, as well as social and family characteristics. The descriptive statistics of the dependent, explanatory, and control variables are shown in Table 1. The study sample comprises 1,872 respondents from the CGSS working population. Nearly half of respondents were male (47.1%, *n* = 881) with a mean age of 41.6 years (SD = 12.14, range of 18–77 years), and the vast majority were married (72.9%, *n* = 1,364). Educational attainment varied across the sample (*M* = 7.19, SD = 3.54, range of 1–13), spanning no formal education to postgraduate qualifications.

**Table 1:** Descriptive statistics.

Variable	Meaning	Obs.	Mean	Std. Dev.	Min.	Max.
Dependent variable						
Depression	Level of perceived depression, 1-5 levels	1,870	1.879	0.941	1	5
Explanatory variables						
Work-to-family conflict	Level of work interference with family, 1–5 levels	1,872	1.955	1.053	1	5
Family-to-work conflict	Level of family interference with work, 1–5 levels	1,870	1.598	0.784	1	5
Control variables						
Demographic and human capital characteristics						
Age	Age	1,872	41.600	12.140	18	77
Age squared	Squared term of age/100	1,872	18.780	10.700	3.240	59.290
Gender	Male = 1, Female = 0	1,872	0.471	0.499	0	1
Whether married	Yes = 1, No = 0	1,872	0.729	0.445	0	1
Whether Hukou in urban	Yes = 1, No = 0	1,855	0.405	0.491	0	1
Education level	1–13 levels	1,867	7.194	3.538	1	13
Whether migrant	Yes = 1, No = 0	1,869	0.196	0.397	0	1
Health level	Self-reported health level, 1-5 levels	1,872	3.851	0.864	1	5
Social and family characteristics						
ln_Income	Logarithm of personal income (RMB)	1,872	10.410	2.232	0	16.019
Whether working in the system	Yes = 1, No =0	1,834	0.201	0.401	0	1
Whether ethnic minorities	Yes = 1, No = 0	1,872	0.052	0.222	0	1
Whether CPC member	Yes = 1, No = 0	1,870	0.164	0.371	0	1
Whether religious believer	Yes = 1, No = 0	1,872	0.057	0.231	0	1
Whether having pension	Yes = 1, No = 0	1,871	0.796	0.403	0	1
Whether having medical insurance	Yes = 1, No = 0	1,872	0.965	0.184	0	1
Number of houses	Number of houses in the family	1,856	1.248	0.731	0	11
Family size	Number of family members	1,872	3.264	1.596	1	10
Number of children	Number of children	1,870	1.186	0.928	0	8
Province dummies						

Hukou is a system of household registration used in mainland China, mainly identifying a person as a rural or urban resident. The education level is classified from 1 to 13 (1-without any education, 13-postgraduate and above). Health level is classified from 1 to 5 (1 = very unhealthy, 5 = very healthy). Working in the system in China refers to having jobs in Communist Party of China organizations, governments and state-owned corporations. CPC represents Communist Party of China and CPC member is the most important political identity in China. The dummy variables are all defined as “Yes = 1, No = 0”.

**Figure 1:**
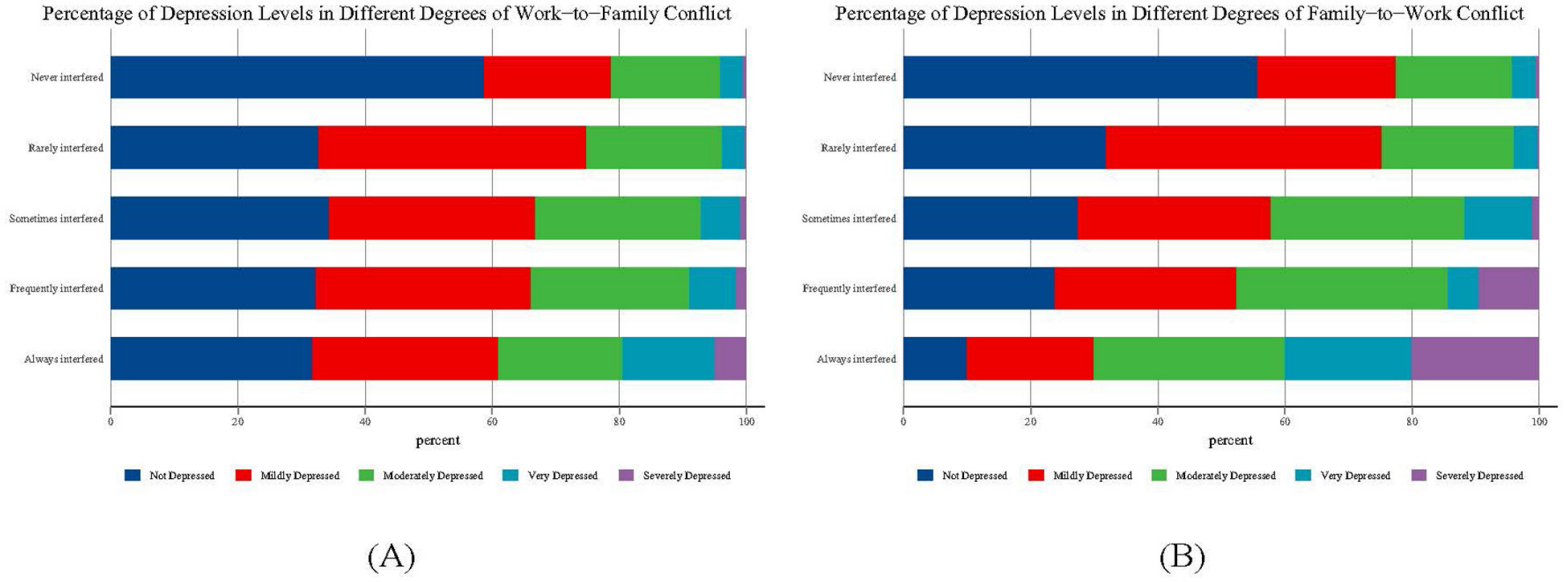
Varied levels of depression across different subgroups of WFC and FWC. **(A)** Percentage of depression levels in different degrees of WFC. **(B)** Percentage of depression levels in different degrees of FWC.

This study conducts a preliminary statistical analysis of the relationship between work-family conflict and perceived depression. Figures 1A, B illustrate the distribution of different levels of perceived depression across subgroups of WFC and FWC. It is illustrated that there is a positive association between work-family conflict and perceived depression, suggesting that as the degree of interference from work or life increases, the level of self-reported depression also rises. Furthermore, FWC's impact on perceived depression appears to be more pronounced. For example, in the “always interfered” group of FWC, severely depressed individuals reach 20.00%, significantly surpassing the corresponding proportion in the “always interfered” group of WFC. The following sections will rigorously examine and analyze the specific relationship between WFC, FWC, and perceived depression.

## 3 Results

### 3.1 Benchmark analysis

Given that the dependent variable in this study is an ordinal indicator rather than a continuous variable, the Ordered Probit model is employed for regression. Specifically, based on the degree of perceived depression, individuals are categorized into five groups. Groups *g* = 1 to 5 represent *Depression*_*i*_ = 1 (not depressed), *Depression*_*i*_ = 2 (mildly depressed), *Depression*_*i*_ = 3 (moderately depressed), *Depression*_*i*_ = 4 (very depressed) and *Depression*_*i*_ = 5 (severely depressed), respectively. The probability of individual *i* in group *g* is denoted as *p*_*gi*_.


$$Pg_{i}=Pr\left(Depression_{i}=g\right)=Pr\left(\chi_{g-1}<\alpha_{0}+\alpha_{1}WFC_{i}+\alpha_{2}FWC_{i}+{x^{\prime }}_{i}\psi^{1}+d_{r}+\varepsilon_{i}^{1}\leq \chi_{g}\right)$$ (1)

$$Pr(.)=\Phi \left(\chi_{g}-\alpha_{0}-\alpha_{1}WFC_{i}-\alpha_{2}FWC_{i}-{x^{\prime }}_{i}\psi^{1}-d_{r}\right)-\Phi \left(\chi_{g}-1-\alpha_{0}-\alpha_{1}WFC_{i}-\alpha_{2}FWC_{i}-{x^{\prime }}_{i}\psi^{1}-d_{r}\right)$$ (2)

*Depression*_*i*_ refers to the respondents' perceived level of depression. The main explanatory variables are *WFC*_*i*_ and *FWC*_*i*_, representing the degree of work-to-family conflict and family-to-work conflict, respectively. ${x^{\prime }}_{i}$ and *d*_*r*_ are the vector of control variables and provincial dummy variables. χ_0_ is −∞ and χ_5_ is +∞. Φ(·) is the standard normal cumulative distribution function. Accordingly, the log-likelihood of the maximum likelihood estimation is


$$\begin{array}{l} lnL=\overset{N}{\underset{i=1}{\sum }}\overset{5}{\underset{g=1}{\sum }}I_{g}\left(Depression_{i}\right)\ln\;p_{gi} \end{array}$$ (3)

where $I_{g}(Depression_{i})=\{\begin{array}{ll} 1 & \;if\;Depression_{i}=g\\ 0 & if\;Depression_{i}\neq g \end{array}$ and *N* is the sample size. Based on this, α_1_, α_2_ and **ψ**^**1**^ are estimated by $\underset{\alpha_{1},\alpha_{2},\psi^{1}}{\max}lnL$.

The estimation results using Ordered Probit model are presented in Table 2. Columns (1) to (3) estimate the relationship between WFC and perceived depression. Column (1) excludes all control variables. The results show that the estimated coefficient of WFC is significantly positive at the 1% level, indicating a positive association between WFC and depression. As control variables related to demographic and human capital characteristics, as well as social and family characteristics, are gradually added in columns (2) and (3), the coefficients for WFC remain significantly positive with little change in magnitude. This suggests that the relationship between WFC and perceived depression is robust and largely unaffected by other factors. Columns (4)–(6) of Table 2 estimate the association between FWC and perceived depression. Regardless of whether and which control variables are included, the estimated coefficient for FWC is significantly positive at the 1% level, and the coefficient is remains stable. This demonstrates the robust relationship between FWC and perceived depression.

**Table 2:** Benchmark regressions.

Model	Statistic	(1)	(2)	(3)	(4)	(5)	(6)	(7)
		Ordered Probit	Ordered Probit	Ordered Probit	Ordered Probit	Ordered Probit	Ordered Probit	Ordered Probit
Variable		Depression	Depression	Depression	Depression	Depression	Depression	Depression
Work-to-family conflict	β	0.209^***^	0.207^***^	0.202^***^				0.104^***^
	SE	(0.026)	(0.028)	(0.029)				(0.033)
	*P*	< 0.001	< 0.001	< 0.001				0.002
	95% CI	[0.158, 0.260]	[0.153, 0.261]	[0.146, 0.258]				[0.039, 0.169]
Family-to-work conflict	β				0.326^***^	0.332^***^	0.331^***^	0.268^***^
	SE				(0.035)	(0.037)	(0.038)	(0.044)
	*P*				< 0.001	< 0.001	< 0.001	< 0.001
	95% CI				[0.258, 0.395]	[0.261, 0.407]	[0.259, 0.407]	[0.181, 0.355]
Age	β		−0.029^*^	−0.029^*^		−0.021	−0.022	−0.023
	SE		(0.016)	(0.017)		(0.016)	(0.017)	(0.017)
	*P*		0.087	0.108		0.160	0.176	0.169
	95% CI		[−0.058, 0.004]	[−0.061, 0.006]		[−0.052, 0.009]	[−0.055, 0.010]	[−0.056, 0.010]
Age squared	β		0.026	0.027		0.016	0.018	0.021
	SE		(0.018)	(0.019)		(0.017)	(0.018)	(0.018)
	*P*		0.172	0.182		0.318	0.318	0.268
	95% CI		[−0.011, 0.059]	[−0.012, 0.062]		[−0.017, 0.051]	[−0.018, 0.054]	[−0.016, 0.057]
Gender	β		0.164^***^	0.160^***^		0.112^**^	0.111^**^	0.138^**^
	SE		(0.053)	(0.056)		(0.053)	(0.056)	(0.056)
	*P*		0.002	0.004		0.029	0.038	0.014
	95% CI		[0.064, 0.271]	[0.053, 0.272]		[0.012, 0.218]	[0.006, 0.224]	[0.028, 0.247]
Whether married	β		−0.154^**^	−0.133^*^		−0.142^**^	−0.133^*^	−0.140^*^
	SE		(0.068)	(0.074)		(0.066)	(0.073)	(0.073)
	*P*		0.020	0.064		0.031	0.068	0.055
	95% CI		[−0.291, −0.025]	[−0.281, 0.008]		[−0.273, −0.013]	[−0.275, 0.010]	[−0.283, 0.003]
Whether Hukou in urban	β		0.029	0.037		0.027	0.038	0.041
	SE		(0.058)	(0.062)		(0.059)	(0.062)	(0.062)
	*P*		0.629	0.583		0.612	0.511	0.507
	95% CI		[−0.086, 0.142]	[−0.087, 0.155]		[−0.085, 0.144]	[−0.081, 0.162]	[−0.081, 0.163]
Education level	β		−0.002	0.015		0.003	0.016	0.015
	SE		(0.009)	(0.011)		(0.009)	(0.011)	(0.011)
	*P*		0.805	0.195		0.732	0.129	0.172
	95% CI		[−0.020, 0.015]	[−0.007, 0.036]		[−0.015, 0.021]	[−0.005, 0.038]	[−0.007, 0.037]
Whether migrant	β		−0.141^**^	−0.136^*^		−0.108^*^	−0.115	−0.125^*^
	SE		(0.065)	(0.073)		(0.065)	(0.072)	(0.073)
	*P*		0.033	0.065		0.098	0.123	0.088
	95% CI		[−0.267, −0.011]	[−0.277, 0.009]		[−0.234, 0.020]	[−0.253, 0.030]	[−0.267, 0.018]
Health level	β		−0.478^***^	−0.471^***^		−0.495^***^	−0.487^***^	−0.483^***^
	SE		(0.035)	(0.037)		(0.035)	(0.036)	(0.037)
	*P*		< 0.001	< 0.001		< 0.001	< 0.001	< 0.001
	95% CI		[−0.550, −0.411]	[−0.546, −0.402]		[−0.566, −0.429]	[−0.561, −0.418]	[−0.554, −0.411]
ln_Income	β			−0.005			−0.003	−0.007
	SE			(0.014)			(0.014)	(0.014)
	*P*			0.719			0.833	0.617
	95% CI			[−0.032, 0.022]			[−0.030, −0.024]	[−0.034, −0.020]
Whether working in the system	β			0.003			0.006	−0.001
	SE			(0.075)			(0.077)	(0.077)
	*P*			0.968			0.938	0.990
	95% CI			[−0.144, 0.150]			[−0.145, 0.157]	[−0.152, 0.150]
Whether ethnic minorities	β			0.203			0.197	0.191
	SE			(0.144)			(0.146)	(0.146)
	*P*			0.159			0.177	0.191
	95% CI			[−0.079, 0.485]			[−0.089, 0.483]	[−0.095, 0.477]
Whether CPC member	β			−0.166^**^			−0.134^*^	−0.144^*^
	SE			(0.079)			(0.080)	(0.080)
	*P*			0.035			0.093	0.072
	95% CI			[−0.321, −0.011]			[−0.291, 0.023]	[−0.301, 0.013]
Whether religious believer	β			−0.184			−0.188	−0.181
	SE			(0.125)			(0.124)	(0.125)
	*P*			0.141			0.129	0.147
	95% CI			[−0.429, 0.061]			[−0.431, 0.055]	[−0.426, 0.064]
Whether having pension	β			−0.107			−0.088	−0.092
	SE			(0.074)			(0.073)	(0.074)
	*P*			0.147			0.228	0.213
	95% CI			[−0.252, 0.038]			[−0.231, 0.055]	[−0.237, 0.053]
Whether having medical insurance	β			−0.047			−0.043	−0.051
	SE			(0.181)			(0.173)	(0.175)
	*P*			0.795			0.803	0.771
	95% CI			[−0.401, 0.307]			[−0.382, 0.296]	[−0.394, 0.292]
Number of houses	β			−0.036			−0.033	−0.030
	SE			(0.041)			(0.040)	(0.040)
	*P*			0.379			0.408	0.453
	95% CI			[−0.117, 0.045]			[−0.111, 0.045]	[−0.108, 0.048]
Family size	β			−0.035^*^			−0.036^**^	−0.037^**^
	SE			(0.019)			(0.018)	(0.018)
	*P*			0.065			0.046	0.039
	95% CI			[−0.072, 0.002]			[−0.071, −0.001	[−0.072, −0.002]
Number of children	β			0.085^**^			0.092^**^	0.087^**^
	SE			(0.037)			(0.037)	(0.037)
	*P*			0.022			0.013	0.019
	95% CI			[0.012, 0.158]			[0.019, 0.165]	[0.014, 0.160]
Province Dummies		No	No	Yes	No	No	Yes	Yes
Observations		1,870	1,847	1,794	1,869	1,846	1,793	1,792
Pseudo *R*^2^		0.016	0.071	0.080	0.022	0.079	0.087	0.091

^***^, ^**^, and ^*^ indicate significance at the levels of 1%, 5%, and 10%, respectively. The values in parentheses are standard errors robust to heteroscedasticity. CI refers to the confidence interval. Yes means the corresponding variables are controlled, while No means not controlled. Pseudo *R*^2^ is reported for non-linear models.

**Figure 2:**
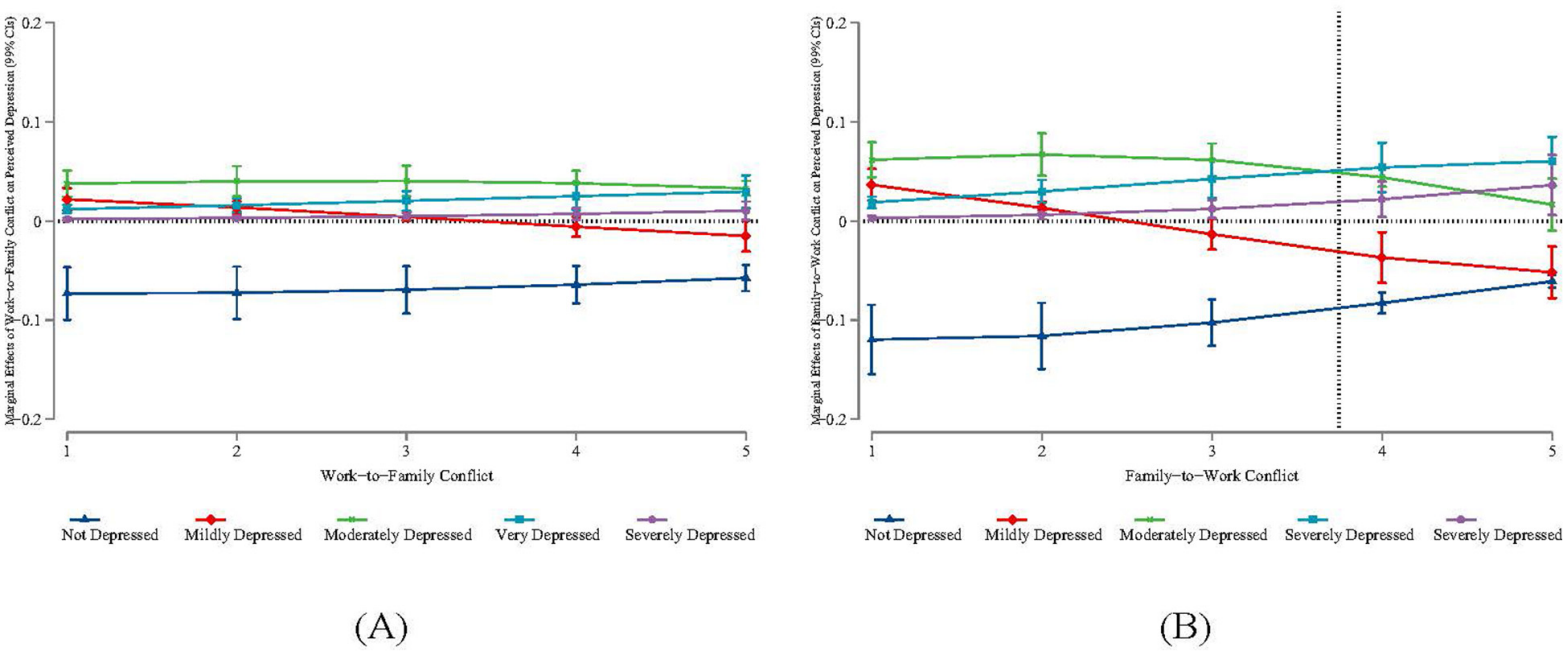
Marginal effects of WFC and FWC on perceived depression. **(A)** Marginal effects of WFC on perceived depression. **(B)** Marginal effects of FWC on perceived depression.

Furthermore, when both WFC and FWC are included in the regression equation, the results in column (7) show that their relationships with perceived depression remain significantly positive at the 1% level. This indicates that both aspects of work-family conflict independently explain perceived depression and are critical correlates thereof. In addition, the coefficient for FWC (0.268) is about 2.5 times that of WFC (0.104), indicating that FWC tends to exert a stronger effect on perceived depression. Given that both depression and conflict are measured on 1–5 Likert scales, a one-unit increase in FWC is associated with a higher latent propensity to report stronger depressive symptoms, which, in the ordered probit framework, shifts the probability mass toward higher depression categories. By contrast, a one-unit increase in WFC corresponds to a 0.104 increase in the latent index, indicating a weaker shift toward higher depression categories. Overall, the results suggest that FWC is more strongly associated with higher perceived depression levels than WFC. This difference may stem from the emphasis on work in the Chinese tradition, where diligence is often considered a virtue. The societal focus on work makes people prioritize work over family when facing conflict (45). This finding is consistent with the preliminary results above, suggesting that FWC is more strongly associated with perceived depression than WFC. This study further visualizes the marginal effects of WFC and FWC on perceived depression in Figures 2A, B, respectively. It intuitively demonstrates that although both elevate the risk of higher depression levels, the marginal effects of FWC are greater than that of WFC. Moreover, robustness checks using different regression models and depression indicators are presented in the Supplementary materials, confirming the main findings.

### 3.2 Mechanism analysis

#### 3.2.1 Life happiness mechanism

This study further explores the underlying mechanisms by which WFC and FWC influence perceived depression. Previous research shows that work-family conflict significantly reduces subjective life happiness (20, 21). Additionally, lower happiness is often associated with a higher risk of depression (38). Building upon this foundation, we hypothesize that work-family conflict may operate indirectly on depression by attenuating life happiness. This indicator is derived from the question in CGSS: “Overall, do you feel happy in your life?” Responses range from 1 (very unhappy) to 5 (very happy), with higher scores indicating greater life happiness. Results in column (2) of Table 3 provide support for our hypothesis. Both WFC and FWC exhibit substantial negative associations with happiness, with coefficients of −0.080 (*p* = 0.082) and −0.243 (*p* < 0.001), respectively. This indicates that individuals experiencing elevated work-family pressures report markedly lower life happiness. Column (3) indicates a significantly negative relationship between happiness and perceived depression. Thus, WFC and FWC increase depression by reducing life happiness. To test the robustness of this mechanism, the study constructs a dummy variable for happiness, Whe_Happiness, which equals 1 when Happiness is 4 (happy) or 5 (very happy) and 0 otherwise. Results using Whe_Happiness in columns (4) and (5) of Table 3 show that happiness mediates the effects of WFC and FWC on self-rated depression, demonstrating the robustness of this mechanism.

**Table 3:** Life happiness mechanism of WFC and FWC on perceived depression.

Model	Statistic	(1)	(2)	(3)	(4)	(5)
		Ordered Probit	Ordered Probit	Ordered Probit	Ordered Probit	Ordered Probit
Variable		Depression	Happiness	Depression	Whe_Happiness	Depression
Work-to-family conflict	β	0.104^***^	−0.080^*^	0.142^***^	−0.103^*^	0.149^***^
	SE	(0.033)	(0.046)	(0.040)	(0.053)	(0.041)
	*P*	0.002	0.082	< 0.001	0.052	< 0.001
	95% CI	[0.039, 0.169]	[−0.171, 0.010]	[0.063, 0.221]	[−0.209, 0.001]	[0.068, 0.230]
Family-to-work conflict	β	0.268^***^	−0.243^***^	0.184^***^	−0.271^***^	0.200^***^
	SE	(0.044)	(0.060)	(0.052)	(0.067)	(0.054)
	*P*	< 0.001	< 0.001	< 0.001	< 0.001	< 0.001
	95% CI	[0.181, 0.355]	[−0.358, −0.123]	[0.082, 0.285]	[−0.399, −0.135]	[0.095, 0.306]
Happiness	β			−0.298^***^		
	SE			(0.055)		
	*P*			< 0.001		
	95% CI			[−0.406, −0.191]		
Whe_Happiness	β					−0.349^***^
	SE					(0.095)
	*P*					< 0.001
	95% CI					[−0.536, −0.163]
Control variables		Yes	Yes	Yes	Yes	Yes
Observations		1,792	1,162	1,160	1,162	1,160
Pseudo *R*^2^		0.091	0.099	0.104	0.171	0.096

^***^, ^**^, and ^*^ indicate significance at the levels of 1%, 5%, and 10%, respectively. The values in parentheses are standard errors robust to heteroscedasticity. CI refers to the confidence interval. Yes means the corresponding variables are controlled, while No means not controlled. Pseudo *R*^2^ is reported for non-linear models.

#### 3.2.2 Work satisfaction mechanism

Previous research reveals that work-family conflict negatively affects work satisfaction (36, 37). Additionally, work satisfaction is negatively associated with depression (38). As a result, this study further investigates whether WFC and FWC increase perceived depression by lowering work satisfaction. Work satisfaction is measured using respondents' answers to the question “Are you satisfied with your current job?” Responses are on a five-point Likert scale, with higher scores indicating greater satisfaction. To test the robustness of this mechanism, a dummy variable for work satisfaction, Whe_Satisfaction, is also constructed. Whe_Satisfaction =1 when work satisfaction is 4 (satisfied) or 5 (very satisfied) and 0 otherwise. Table 4 presents the results of this mechanism analysis. Column (2) reveals significant negative coefficients for both WFC (β = −0.280, *p* < 0.001) and FWC (β = −0.079, *p* = 0.067), indicating that work-family conflicts substantially reduce respondents' work satisfaction. Notably, WFC exerts a substantially stronger effect on work satisfaction compared to FWC, suggesting that work pressures more directly undermine work satisfaction. Column (4) confirms this same pattern. Columns (3) and (5) further indicate that work satisfaction and Whe_Satisfaction yield significantly negative coefficients of −0.229 and −0.282 respectively. When work satisfaction is incorporated, the direct coefficients of WFC and FWC on depression exhibit partial attenuation. Therefore, this provides evidence consistent with the view that both WFC and FWC increase perceived depression by reducing work satisfaction. These results demonstrate the robustness and reliability of this conclusion.

**Table 4:** Work satisfaction mechanism of WFC and FWC on perceived depression.

Model	Statistic	(1)	(2)	(3)	(4)	(5)
		Ordered Probit	Ordered Probit	Ordered Probit	Ordered Probit	Ordered Probit
Variable		Depression	Work Satisfaction	Depression	Whe_Satisfaction	Depression
Work-to-family conflict	β	0.104^***^	−0.280^***^	0.059^*^	−0.280^***^	0.078^**^
	SE	(0.033)	(0.032)	(0.035)	(0.037)	(0.034)
	*P*	0.002	< 0.001	0.091	< 0.001	0.022
	95% CI	[0.039, 0.170]	[−0.344, −0.217]	[−0.011, 0.128]	[−0.353, −0.207]	[0.011, 0.145]
Family-to-work conflict	β	0.268^***^	−0.079^*^	0.255^***^	−0.091^*^	0.254^***^
	SE	(0.044)	(0.043)	(0.044)	(0.049)	(0.045)
	*P*	< 0.001	0.067	< 0.001	0.063	< 0.001
	95% CI	[0.182, 0.355]	[−0.162, −0.005]	[0.169, 0.342]	[−0.187, 0.005]	[0.166, 0.342]
Work satisfaction	β			−0.229^***^		
	SE			(0.039)		
	*P*			< 0.001		
	95% CI			[−0.306, −0.152]		
Whe_Satisfaction	β					−0.282^***^
	SE					(0.060)
	*P*					< 0.001
	95% CI					[−0.399, −0.165]
Control variables		Yes	Yes	Yes	Yes	Yes
Observations		1,792	1,789	1,787	1,789	1,787
Pseudo *R*^2^		0.091	0.080	0.100	0.121	0.095

^***^, ^**^, and ^*^ indicate significance at the levels of 1%, 5%, and 10%, respectively. The values in parentheses are standard errors robust to heteroscedasticity. CI refers to the confidence interval. Yes means the corresponding variables are controlled, while No means not controlled. Pseudo *R*^2^ is reported for non-linear models.

### 3.3 Heterogeneity analysis

#### 3.3.1 Heterogeneity in terms of demographic characteristics

Previous analysis finds that WFC and FWC significantly increase perceived depression. However, due to individual differences, the degree of this impact may vary. Therefore, this study examines heterogeneities by demographic characteristics such as gender, age and education level. Regarding gender (Table 5, columns 1–2), WFC exhibits a markedly stronger effect on men (β = 0.153, *p* < 0.001) compared to women (β = 0.045, *p* = 0.434). This disparity may stem from traditional gender roles, where men typically bear more economic responsibilities in the family (46). Thus, when work interferes with family life, they experience more negative emotions and stress. However, FWC's impact shows no significant gender difference, consistent with existing literature (47, 48). In terms of age, columns (3) and (4) show that the association between WFC and perceived depression is stronger for individuals under 40 (β = 0.181, *p* = 0.002) than for those above 40 (β = 0.056, *p* = 0.109). This may be because younger people are at a critical stage in their careers, encountering greater development pressures and challenges, thus becoming more prone to WFC. In contrast, FWC's impact on depression does not vary significantly across different age groups. Regarding education level, the sample is categorized into higher and lower educated groups based on whether respondents have a college degree or higher. The results in columns (5) and (6) indicate that WFC has a greater impact on perceived depression among people with higher education. This could be attributed to their elevated expectations for career advancement (49), making it easier for work to interfere with their family life and consequently leading to depression. However, FWC affects self-rated depression more among the lower educated population. This may be because they typically engage in less flexible jobs (50), which limits their ability to adjust work schedules for family demands (51), potentially increasing the risk of depression.

**Table 5:** Heterogeneity in terms of demographic characteristics (Ordered Probit).

Sample	Statistic	(1)	(2)	(3)	(4)	(5)	(6)
		Men	Women	Younger than 40	Older than 40	Lower education levels	Higher education levels
Variable		Depression	Depression	Depression	Depression	Depression	Depression
Work-to-family conflict	β	0.153^***^	0.045	0.181^***^	0.056	0.081^*^	0.157^***^
	SE	(0.041)	(0.058)	(0.053)	(0.044)	(0.047)	(0.050)
	*P*	< 0.001	0.434	0.002	0.109	0.037	0.007
	95% CI	[0.073, 0.234]	[−0.068, 0.158]	[0.056, 0.258]	[−0.016, 0.158]	[0.005, 0.173]	[0.041, 0.264]
Family-to-work conflict	β	0.271^***^	0.274^***^	0.278^***^	0.250^***^	0.313^***^	0.186^***^
	SE	(0.058)	(0.071)	(0.071)	(0.056)	(0.061)	(0.065)
	*P*	< 0.001	< 0.001	< 0.001	< 0.001	< 0.001	0.036
	95% CI	[0.157, 0.384]	[0.134, 0.414]	[0.170, 0.439]	[0.117, 0.344]	[0.205, 0.421]	[0.010, 0.309]
Control variables		Yes	Yes	Yes	Yes	Yes	Yes
Observations		946	846	905	887	1,047	745
Pseudo *R*^2^		0.122	0.086	0.119	0.081	0.104	0.091

^***^, ^**^, and ^*^ indicate significance at the levels of 1%, 5%, and 10%, respectively. The values in parentheses are standard errors robust to heteroscedasticity. CI refers to the confidence interval. Yes means the corresponding variables are controlled, while No means not controlled. Pseudo *R*^2^ is reported for non-linear models.

#### 3.3.2 Heterogeneity in terms of family characteristics

Family dynamics play a crucial role in work-family conflict. Thus, this study analyzes heterogeneity in terms of family characteristics along three dimensions: marital status, whether or not having children, and family economic status. First, regarding marital status, subsample regressions are conducted for married and unmarried groups, as shown in columns (1) and (2) of Table 6. The results reveal that WFC exerts substantially greater influence on perceived depression among married respondents (β = 0.158, *p* < 0.001) compared to unmarried respondents (β = 0.010, *p* = 0.892). This is because married ones typically take on more family responsibilities, so work is more likely to affect their emotional well-being (52). Conversely, FWC has a slightly larger impact on unmarried ones, possibly due to the absence of a partner to share family pressures, resulting in increased stress when family obligations interfere with work. Moreover, childrearing constitutes a central dimension of family life. Accordingly, subsample regressions are performed based on whether respondents have children. Results presented in columns (3) and (4) reveal that WFC exerts a stronger influence on perceived depression among individuals with children (β = 0.124, *p* = 0.002) than among those without children (β = 0.083, *p* = 0.238). FWC shows a similar pattern, with stronger effects among parents (β = 0.273, *p* < 0.001) than non-parents (β = 0.246, *p* = 0.013). This implies that WFC and FWC have more prominent effects among groups with children, consistent with existing literature (53). This may be due to the fact that individuals having children allocate more time and resources to childrearing, intensifying the trade-off between work and family, thereby increasing perceived depression. Lastly, concerning family economic status, columns (5) and (6) demonstrate that the impact of WFC and FWC on perceived depression is more substantial among lower-income people. This disparity may stem from the fact that limited economic resources restrict access to social support and family services for individuals with lower family economic status (51), which could help mitigate the conflict between work and family responsibilities.

**Table 6:** Heterogeneity in terms of family characteristics (Ordered Probit).

Sample	Statistic	(1)	(2)	(3)	(4)	(5)	(6)
		Unmarried	Married	Having no children	Having children	Lower family economic status	Higher family economic status
Variable		Depression	Depression	Depression	Depression	Depression	Depression
Work-to-family conflict	β	0.010	0.158^***^	0.083	0.124^***^	0.098^***^	0.101
	SE	(0.066)	(0.039)	(0.070)	(0.039)	(0.036)	(0.119)
	*P*	0.892	< 0.001	0.238	0.002	0.001	0.391
	95% CI	[−0.121, 0.138]	[0.082, 0.234]	[−0.054, 0.219]	[0.046, 0.199]	[0.066, 0.249]	[−0.131, 0.335]
Family-to-work conflict	β	0.393^***^	0.235^***^	0.246^**^	0.273^***^	0.294^***^	0.104
	SE	(0.090)	(0.049)	(0.099)	(0.050)	(0.047)	(0.165)
	*P*	< 0.001	< 0.001	0.013	< 0.001	0.006	0.529
	95% CI	[0.217, 0.570]	[0.139, 0.331]	[0.052, 0.441]	[0.176, 0.372]	[0.044, 0.272]	[−0.219, 0.427]
Control variables		Yes	Yes	Yes	Yes	Yes	Yes
Observations		479	1,313	419	1,373	1,594	169
Pseudo *R*^2^		0.113	0.101	0.104	0.096	0.089	0.234

^***^, ^**^, and ^*^ indicate significance at the levels of 1%, 5%, and 10%, respectively. The values in parentheses are standard errors robust to heteroscedasticity. CI refers to the confidence interval. Yes means the corresponding variables are controlled, while No means not controlled. Pseudo *R*^2^ is reported for non-linear models.

#### 3.3.3 Heterogeneity in terms of work characteristics

This study further investigates the heterogeneity of work-family conflict across various work attributes. First, the sample is stratified by respondents' managerial status. Results in columns (1) and (2) of Table 7 reveal that WFC has a stronger effect on individuals in managerial positions (β = 0.262, *p* < 0.001) compared to non-managerial workers (β = 0.055, *p* = 0.179). This heightened effect may be attributed to the increased job responsibilities among managers, such as complex decision-making and team coordination (51), which may intensify perceived depression. In contrast, there is little difference in the effect of FWC on perceived depression between managerial and non-managerial respondents. Second, regarding job autonomy, columns (3) and (4) reveal that both WFC and FWC significantly elevate perceived depression among individuals with lower job autonomy (WFC: β = 0.110, *p* = 0.002; FWC: β = 0.249, *p* < 0.001). This effect might result from limited flexibility in adjusting work arrangements to cope with family or occupational stressors. Third, considering job nature, WFC exhibits greater potency among individuals in non-cognitive occupations (β = 0.145, *p* < 0.001) relative to cognitive work (β = 0.049, *p* = 0.355). While FWC also proves somewhat more disruptive for non-cognitive workers (β = 0.265, *p* < 0.001) than their cognitive counterparts (β = 0.192, *p* = 0.009), the differential is relatively modest, as shown in columns (5) and (6). This disparity may arise from the greater temporal and spatial flexibility typically linked with cognitive work, coupled with superior working conditions (54), which may facilitate better work-family balance for individuals performing cognitive jobs.

**Table 7:** Heterogeneity in terms of work characteristics (Ordered Probit).

Sample	Statistic	(1)	(2)	(3)	(4)	(5)	(6)
		Non–managerial work	Managerial work	Lower job autonomy	Higher job autonomy	Non–cognitive work	Cognitive work
Variable		Depression	Depression	Depression	Depression	Depression	Depression
Work-to-family conflict	β	0.055	0.262^***^	0.110^***^	0.047	0.145^***^	0.049
	SE	(0.041)	(0.058)	(0.035)	(0.115)	(0.043)	(0.053)
	*P*	0.179	< 0.001	0.002	0.683	< 0.001	0.355
	95% CI	[−0.025, 0.135]	[0.149, 0.376]	[0.041, 0.179]	[−0.178, 0.272]	[0.061, 0.229]	[−0.055, 0.153]
Family-to-work conflict	β	0.285^***^	0.227^***^	0.249^***^	0.262^*^	0.265^***^	0.192^***^
	SE	(0.051)	(0.088)	(0.047)	(0.155)	(0.054)	(0.074)
	*P*	< 0.001	0.01	< 0.001	0.091	< 0.001	0.009
	95% CI	[0.186, 0.385]	[0.055, 0.400]	[0.157, 0.341]	[−0.042, 0.566]	[0.159, 0.371]	[0.047, 0.337]
Control variables		Yes	Yes	Yes	Yes	Yes	Yes
Observations		1,323	466	1,685	104	1,265	517
Pseudo *R*^2^		0.096	0.121	0.090	0.255	0.102	0.099

“Non-cognitive work” refers to jobs with low time–space flexibility, single job content and low requirements for cognitive abilities such as analysis and decision-making. ^***^, ^**^, and ^*^ indicate significance at the levels of 1%, 5%, and 10%, respectively. The values in parentheses are standard errors robust to heteroscedasticity. CI refers to the confidence interval. Yes means the corresponding variables are controlled, while No means not controlled. Pseudo *R*^2^ is reported for non-linear models.

#### 3.3.4 Heterogeneity in terms of labor protection

This study further examines how the effects vary across different labor protection conditions. Regression results in columns (1) and (2) of Table 8 show that both WFC and FWC have more significant effects on employees who are working outside the system, which is regarded as the secondary labor market in China. This suggests that stronger labor protection in the system may help mitigate the impact of work-family conflict on perceived depression. From a social security perspective, columns (3) and (4) show that individuals without pension insurance experience larger effects of both WFC (β = 0.166, *p* = 0.031) and FWC (β = 0.352, *p* < 0.001) compared to those with coverage (β = 0.095, *p* = 0.013 for WFC; β = 0.235, *p* < 0.001 for FWC). This indicates that lacking social security coverage substantially heightens depression risk from work-family conflict. Furthermore, the study explores heterogeneity concerning labor market competition. Provinces are categorized into high and low competition regions based on the median proportion of employed university graduates. Results in columns (5) and (6) demonstrate that in the higher competitive labor market, the impact of WFC on perceived depression is more pronounced (β = 0.117, *p* = 0.007) compared to lower-competition regions (β = 0.097, *p* = 0.072). This may be due to increased work pressures in the competitive market, which may intensify WFC.

**Table 8:** Heterogeneity in terms of labor protection (Ordered Probit).

Sample		(1)	(2)	(3)	(4)	(5)	(6)
		Working outside the system	Working in the system	Without social security	With social security	Regions with lower labor market competition	Regions with higher labor market competition
Variable		Depression	Depression	Depression	Depression	Depression	Depression
Work-to-family conflict	β	0.089^***^	0.427	0.166^**^	0.095^**^	0.097^*^	0.117^***^
	SE	(0.034)	(0.270)	(0.077)	(0.038)	(0.054)	(0.043)
	*P*	0.009	0.112	0.031	0.013	0.072	0.007
	95% CI	[0.022, 0.156]	[−0.102, 0.956]	[0.015, 0.317]	[0.020, 0.170]	[−0.009, 0.203]	[0.033, 0.201]
Family-to-work conflict	β	0.298^***^	0.032	0.352^***^	0.235^***^	0.258^***^	0.274^***^
	SE	(0.045)	(0.391)	(0.104)	(0.048)	(0.071)	(0.056)
	*P*	< 0.001	0.936	< 0.001	< 0.001	< 0.001	< 0.001
	95% CI	[0.210, 0.386]	[−0.734, 0.798]	[0.148, 0.556]	[0.144, 0.329]	[0.119, 0.397]	[0.164, 0.384]
Control variables		Yes	Yes	Yes	Yes	Yes	Yes
Observations		1,717	75	374	1,410	762	1,030
Pseudo *R*^2^		0.093	0.366	0.127	0.090	0.111	0.085

^***^, ^**^, and ^*^ indicate significance at the levels of 1%, 5%, and 10%, respectively. The values in parentheses are standard errors robust to heteroscedasticity. CI refers to the confidence interval. Yes means the corresponding variables are controlled, while No means not controlled. Pseudo *R*^2^ is reported for non-linear models.

## 4 Discussion

This study systematically examines the relationship between work-family conflict and perceived depression by distinguishing between WFC and FWC across heterogeneous groups. Results demonstrate that both WFC and FWC significantly increase perceived depression, with FWC having a more pronounced effect. The conclusions remain robust when using different models and depression indicators. Mechanism analysis reveals that WFC and FWC increase perceived depression by reducing life happiness and work satisfaction. Furthermore, this study explores the heterogeneity in the relationship between work-family conflict and self-reported depression across different subgroups. We find that WFC has a more significant impact among individuals who are male, under 40, with lower education levels, married, having children, and in poorer economic status. Additionally, its effect is more prominent among those in managerial positions, with lower job autonomy, performing non-cognitive tasks, working in the system, without pension insurance, and in regions with higher labor market competition. Compared to WFC, FWC shows a stronger effect among highly educated and unmarried people. However, its impact does not differ significantly in terms of gender, age, or managerial roles. In other aspects, the heterogeneity of FWC's influence is largely consistent with that of WFC.

Some findings in this study corroborate conclusions from existing literature. Previous research finds that conflicts between work and family roles lead to work-family balance issues (55, 56), which are closely associated with negative emotions such as depression, anxiety, and burnout (32). This study demonstrates a positive correlation between work-family conflict and perceived depression, aligning with and reinforcing established findings (31, 35). Moreover, by distinguishing between WFC and FWC, this study examines their heterogeneous effects on perceived depression, revealing that FWC has a more pronounced effect. This result deepens our understanding of how work-family conflicts relate to perceived depression.

Furthermore, the mechanism analysis in this study enhances our understanding of the pathways through which work–family conflict is associated with perceived depressive symptoms. Current research on how work-family conflict influences depression remains limited, with a few studies identifying sleep disturbances as a significant mediator (57). This study expands this research area by exploring the roles of life happiness and work satisfaction in the relationship between work-family conflict and depression. The results indicate that work-family conflict increase depression levels by reducing life happiness and work satisfaction. This conclusion advances our comprehension of the mechanisms linking work-family conflict and perceived depression. Besides, the mechanism analysis validates findings from some existing literature. Studies suggest that work-family conflict is negatively correlated with happiness and work satisfaction (58). In addition, other studies reveal that reduced happiness and work satisfaction can lead to depression (59). The mechanism analysis in this study largely supports both strands of the literature.

Moreover, this study's heterogeneity analysis significantly enriches our understanding of how WFC and FWC differentially impact various subgroups. Most literature on the effects of work-family conflict on depression emphasizes gender differences. For instance, research has identified significant gender disparities in the relationship between work-family conflict and mental disorders such as depression and anxiety (60–62). This aligns with our finding that there is gender heterogeneity in the impact of WFC. We also find that FWC affects depression similarly in men and women, consistent with other studies' findings (48). Additionally, previous research highlights the influence of work characteristics, such as work hours and conditions (63, 64). Our study expands on this by investigating how other work-related factors influence the relationship between work-family conflict and perceived depression. Results show that work-family conflict more strongly affects individuals with lower job autonomy and those in non-cognitive occupations. Moreover, this study also examines variations in WFC's impact according to managerial roles. Furthermore, it reveals how the effects of WFC and FWC differ across different demographic, family, work, and labor protection factors.

Based on these findings, reducing work-family conflict may also generate economic beyond individual mental health. Specifically, since WFC and FWC operate through diminished life happiness and work satisfaction, interventions that alleviate such conflict are expected to lower depressive symptoms. This, in turn, may reduce absenteeism, presentism, and turnover intention, and help sustain employee engagement and job performance. From an organizational perspective, such improvements can translate into more stable staffing, lower recruitment and training costs, and fewer disruptions caused by workplace stress and withdrawal behaviors. Future researchers can further examine the linkage between reducing work-family conflict and organizational efficiency, as well as potential cost savings.

## 5 Conclusion

### 5.1 Main findings

This study demonstrates a significant association between work-family conflict and perceived depression in the Chinese workforce. Life happiness and work satisfaction mediate this relationship. Heterogeneous group characteristics moderate the impacts of WFC and FWC. FWC exerts a more pronounced negative effect on depressive symptoms, whereas WFC shows more detrimental effects among specific demographic, family, work, and labor-protection subgroups. These findings highlight the need for targeted interventions that alleviate both WFC and FWC through flexible work arrangements, improved labor protection, and group-specific mental health support.

### 5.2 Implications for practice

This study offers significant policy implications for understanding the relationship between WFC/FWC and perceived depression, and for improving mental health. First, when assessing risk factors for depression, it is important to consider the differential impacts of WFC and FWC. The study highlights that FWC has a more pronounced effect on depression. This suggests that policies should address not only traditional work-related stress but also the ways in which family responsibilities interfere with work. For instance, offering high-quality, affordable childcare and eldercare, paired with strong family leave policies that provide sufficient paid leave for caregiving, can reduce FWC's impact and thereby lower the risk of depression. Second, attention should be given to the heterogeneous effects of WFC and FWC on depression across different groups. Policies should be targeted to high-risk groups, such as younger individuals, those with lower education levels, married people, those with children, and individuals in poorer economic conditions, to mitigate perceived depression associated with work-family conflict. Third, efforts should focus on improving working conditions as well as enhancing labor protection and social security systems. For example, we find that increasing job autonomy could provide workers with more flexibility to balance work and family responsibilities. In addition, stronger labor protection and better social security could help employees receive necessary support and protection when facing conflicts between work and family responsibilities, thereby reducing the impact of work-family conflict on depressive emotions.

Moreover, the findings can inform training programs for HR professionals, managers, and mental health practitioners. Training can emphasize interventions targeting the mediating mechanisms by enhancing employees' life happiness and work satisfaction, thereby buffering the mental health risks associated with work-family conflict. Curricula can draw on the heterogeneity results to help managers and mental health practitioners identify high-risk subgroups. This can support the design of targeted flexibility policies, such as more predictable scheduling and childcare support. It can also enable trainees to implement differentiated support strategies, including targeted psychological support services and employee assistance resources, rather than relying on a one-size-fits-all approach.

### 5.3 Limitations and future research directions

This study has several limitations that should be addressed in future research. First, the study employed a cross-sectional design. As a result, the temporal sequence and dynamic lagged effects remain unclear. Equally importantly, this cross-sectional approach does not account for unmeasured confounders, including personality traits and prior mental health history. These factors may independently predict both exposure to work–family conflict and individuals' depressive symptoms. Future studies should adopt longitudinal or panel designs with repeated measurements to clarify temporal sequencing, estimate lagged effects, and address these unmeasured confounders. Second, our reliance on a single-item measure of perceived depression may pose measurement concerns. This choice introduces the potential for measurement bias and could affect the accuracy of the observed relationships. To improve measurement quality, future work should use standardized, multi-item scales. Third, all data were collected in Chinese settings. Consequently, the findings may have limited generalizability to other cultural or institutional contexts. Specific cultural or institutional factors may shape the heterogeneous impacts of WFC and FWC and the underlying mechanisms. Therefore, future research should broaden its scope and conduct cross-cultural comparisons to test the robustness of these relationships.

## Data Availability

The original contributions presented in the study are included in the article/Supplementary material, further inquiries can be directed to the corresponding authors.
