# Supplementary material for: Heterogeneous impacts of work–family conflict on workforce mental health: evidence from the differential effects of work-to-family and family-to-work conflict
Source: Front Public Health. 2026 May 28;14:1832589. doi: 10.3389/fpubh.2026.1832589 (PMC13253471; doi:10.3389/fpubh.2026.1832589)
Supplement: Supplementary file 1 [file Table_1.docx]

Supplementary Material

# Robustness Checks

## A.1. Robustness checks: using the ordered logit model

In the benchmark regression, this study estimates the function $Pr\left( \cdot\right)$ of perceived depression based on the assumption of a standard normal distribution, thereby constructing the Ordered Probit model. To further examine the robustness of the relationship between WFC and FWC with perceived depression, a $Pr\left( \cdot\right)$ function based on the Logit distribution is employed as shown in model Eq. (A.1), applying the Ordered Logit model for estimation.

| $Pr\left( \cdot\right)=1/\{1+exp(\alpha_{0}+\alpha_{1}{WFC}_{i}+\alpha_{2}{FWC}_{i}+\boldsymbol{x}_{\boldsymbol{i}}^{'}\boldsymbol{\psi}^{\boldsymbol{1}}+d_{r}-\chi_{g})\}-1/\{1+exp(\alpha_{0}+\alpha_{1}{WFC}_{i}+\alpha_{2}{FWC}_{i}+\boldsymbol{x}_{\boldsymbol{i}}^{'}\boldsymbol{\psi}^{\boldsymbol{1}}+d_{r}-\chi_{g-1})\}$ | Eq.(A.1) |
| --- | --- |

Table A.1 presents the results using the Ordered Logit model. Columns (1)-(3) and columns (4)-(6) respectively test the effects of WFC and FWC on perceived depression. Columns (1) and (4) indicate that without any control variables, the coefficients for both WFC and FWC are significantly positive at the 1% level. Columns (2)-(3) and (5)-(6) gradually add two categories of control variables, and the significance of the results remains stable. When WFC and FWC are included in the regression, the results in column (7) show that they both significantly increase perceived depression, and the impact of FWC is more pronounced. This finding corroborates the earlier analysis, further demonstrating that the relationship between WFC, FWC, and perceived depression is highly robust and not dependent on a specific statistical model.

**Table A.1** Using the Ordered Logit Model

| Model | (1)  Ordered Logit | (2)  Ordered Logit | (3)  Ordered Logit | (4)  Ordered Logit | (5)  Ordered Logit | (6)  Ordered Logit | (7)  Ordered Logit |
| --- | --- | --- | --- | --- | --- | --- | --- |
| Variable | Depression | Depression | Depression | Depression | Depression | Depression | Depression |
| Work-to-family conflict | 0.362^***^  (0.045) | 0.357^***^  (0.049) | 0.349^***^  (0.051) |  |  |  | 0.181^***^  (0.058) |
| Family-to-work conflict |  |  |  | 0.575^***^  (0.062) | 0.581^***^  (0.065) | 0.572^***^  (0.067) | 0.458^***^  (0.076) |
| Demographic and Human Capital Characteristics | No | Yes | Yes | No | Yes | Yes | Yes |
| Social and Family Characteristics | No | No | Yes | No | No | Yes | Yes |
| Province Dummies | No | No | Yes | No | No | Yes | Yes |
| Observations | 1870 | 1847 | 1794 | 1869 | 1846 | 1793 | 1792 |
| Pseudo *R*^2^ | 0.017 | 0.071 | 0.080 | 0.023 | 0.079 | 0.088 | 0.091 |

## A.2. Robustness checks: using another perceived depression indicator

This study uses perceived depression as the explanatory variable in the benchmark regression. However, different respondents may interpret the depression levels differently, leading to measurement errors. For example, respondents might have difficulty distinguishing between “very depressed” and “severely depressed”. Two respondents with the same level of depression might report themselves as “very depressed” and “severely depressed”, respectively. To reduce the influence of such potential errors, a dummy variable, Whe_Depression, is constructed to indicate whether a respondent is depressed. Specifically, for the question in the benchmark regression, “To what extent do you feel depressed?” Whe_Depression is assigned a value of 1 if the respondent answers “3-moderately depressed”, “4-very depressed”, or “5-severely depressed”, and 0 otherwise. In the example mentioned above, although individuals may find it challenging to distinguish between “very depressed” and “severely depressed”, their criteria for determining whether they are depressed would not differ significantly. Thus, this variable can largely address the measurement error issue discussed earlier. Based on this dummy variable, a Probit model is used to conduct regression analysis, and the results are shown in Table A.2. The results suggest that regardless of which indicator is used to characterize self-rated depression, the impacts of WFC and FWC on depression are significantly positive. This further confirms the robustness of the results in the benchmark analysis.

**Table A.2** Using another perceived depression indicator

| Model | (1)  Probit | (2)  Probit | (3)  Probit | (4)  Probit | (5)  Probit | (6)  Probit | (7)  Probit |
| --- | --- | --- | --- | --- | --- | --- | --- |
| Variable | Whe_  Depression | Whe_  Depression | Whe_  Depression | Whe_  Depression | Whe_  Depression | Whe_  Depression | Whe_  Depression |
| Work-to-family conflict | 0.150^***^  (0.029) | 0.144^***^  (0.031) | 0.142^***^  (0.032) |  |  |  | 0.071^*^  (0.038) |
| Family-to-work conflict |  |  |  | 0.236^***^  (0.039) | 0.233^***^  (0.042) | 0.239^***^  (0.043) | 0.197^***^  (0.050) |
| Demographic and Human Capital Characteristics | No | Yes | Yes | No | Yes | Yes | Yes |
| Social and Family Characteristics | No | No | Yes | No | No | Yes | Yes |
| Province Dummies | No | No | Yes | No | No | Yes | Yes |
| Constant | -0.939^***^  (0.067) | 1.008^**^  (0.453) | 1.120  (0.722) | -1.026^***^  (0.072) | 0.793^*^  (0.455) | 1.149  (0.711) | 1.069  (0.716) |
| Observations | 1870 | 1847 | 1794 | 1869 | 1846 | 1793 | 1792 |
| Pseudo *R*^2^ | 0.012 | 0.092 | 0.105 | 0.017 | 0.097 | 0.111 | 0.114 |
